# Chronic hepatitis B carriers with acute on chronic liver failure show increased HBV surface gene mutations, including immune escape variants

**DOI:** 10.1186/s12985-017-0870-x

**Published:** 2017-10-24

**Authors:** Shan Gao, Shivali S. Joshi, Carla Osiowy, Y. Chen, Carla S. Coffin, Z-P. Duan

**Affiliations:** 1 Artificial Liver Center, Beijing Youan Hospital, Capital Medical University, 8 Xitoutiao, Youwai Street, Beijing, 100069 China; 2 Calgary Liver Unit, Division of Gastroenterology and Hepatology, Department of Medicine, Cumming School of Medicine, University of Calgary 6D21, Teaching, Research and Wellness Building, 3280 Hospital Drive N.W, Calgary, AB T2N 4Z6 Canada; 3 Department of Microbiology, Immunology and Infectious Diseases, Cumming School of Medicine, University of Calgary, Calgary, AB Canada; 4 Bloodborne Pathogens and Hepatitis Laboratory of the National Microbiology Laboratory, Winnipeg, MB Canada

**Keywords:** Hepatitis B virus, Mutants, Fulminant liver failure, Viral diversity, Immune escape

## Abstract

**Background:**

The pathogenesis of acute-on-chronic liver failure (ACLF) in chronic hepatitis B (CHB) is not well understood. The aim of this study was to investigate whether there is an association between HBV polymerase (P)/overlapping surface (S) gene and basal core promoter (BCP)/precore (PC) variants and development of ACLF in CHB.

**Methods:**

Two CHB patient cohorts were compared: (i) ACLF (*N* = 12) (11/12 M, median age 52 yrs., 5/9 genotype C, 6/12 HBeAg+), (ii) 27 treatment native CHB carriers (15/27 M, median age 44 yrs., 9 genotype B, 10 genotype C, 1 genotype A, 5 genotype D, 2 genotype E). Clonal sequencing of PCR-amplified HBV P/S and BCP/PC gene fragments was done and HBV diversity, frequency of immune escape (IE) and drug resistance (DR) mutations and mutations in BCP/PC gene (G1896A and A1762T/G1764A), were compared between each group.

**Results:**

Our data showed the incidence of IE and clusters of mutations in the HBV S region was significantly greater in ACLF patients vs. treatment naïve CHB patients (*p* < 0.05). Additionally, a significantly higher frequency of G1896A and A1762T/G1764A mutations were found in HBeAg negative than in ACLF patients (*p* < 0.0001).

**Conclusion:**

In our study, ACLF was not associated with a specific genomic mutation. However, higher frequency of IE mutations along with various mutations clustering in the HBV S region could contribute to or be an outcome of ACLF in CHB infection. (words 226).

**Electronic supplementary material:**

The online version of this article (10.1186/s12985-017-0870-x) contains supplementary material, which is available to authorized users.

## Background

The Hepatitis B virus (HBV) is as a non-cytopathic virus and its induced liver injury in chronic hepatitis B (CHB) carriers is mediated by the host immune response. Acute-on-chronic liver failure (ACLF) is defined as an acute hepatic insult manifesting as jaundice and coagulopathy, complicated within 4 weeks by ascites and/or encephalopathy in a patient with chronic liver disease [1]. Severe HBV flares can lead to ACLF, especially in patients with underlying cirrhosis and manifests as sudden HBV DNA increase followed by biochemical (alanine amino transferase, ALT) increase. Acute exacerbation of CHB can affect 40–50% HBeAg positive patients in the immune clearance phase [2]. HBV reactivation is seen in 15–30% of HBeAg negative patients, which can also result in liver disease decompensation [3]. ACLF is associated with >50% 3-month mortality rates without liver transplantation [4, 5], yet the pathogenesis is poorly understood.

The development of ACLF is attributed to both host and viral factors. The viral factors include genotypes, HBe antigen (HBeAg) negativity, and mutations in the HBV precore and core promoter region [6]. High replication of certain viral variants have been associated with a more aggressive disease course [7, 8]. Acute HBV infection with genotype B1 was reported to frequently lead to fulminant hepatitis and a higher mortality rate [9], possibly due to the more immunogenic nature of HBV genotype B and resulting immune-mediated damage [10]. The G1896A mutation in the precore region, which post-transcriptionally abrogates HBeAg expression, was associated with fulminant hepatitis B development [11–13]. The A1762T/G1764A dual mutations in core promoter region were also implicated in fulminant hepatitis [6, 14–16] due to improved transcription of pregenomic (pg)-RNA and increased viral replication. In addition, mutations such as T1753 V (genotype A, C or G), T1754S (genotype C or G), G1862 T, T1961 V, C1962D (A, G or T), and A2339G were more frequently found in fulminant hepatitis [15, 17, 18]. The presence of various mutations clustering at enhancer Π core promoter region was also demonstrated to contribute to the development of fulminant hepatitis [19]. In contrast, other studies found that the presence of precore and core promoter mutations were not associated with a worse prognosis [20] or pathogenesis of fulminant hepatitis B [19]. Additionally, the presence of several naturally occurring S protein mutations in fulminant hepatitis B patients was demonstrated to synergistically affect the secretion competence of HBV, leading to ER stress and hepatocyte injury [21].

Host immunologic factors are key to the pathogenesis of ACLF in CHB [22]. Excessive production of anti-inflammatory cytokines, such as interleukin (IL)-6, IL-10 and IL-12 and interferon-gamma was reported in ACLF-CHB patients due to reduced monocyte activation.

Both host immune response and antiviral selective pressure can promote the emergence of mutations in P/S and BCP/PC regions and evolution of HBV quasispecies. On the other hand, the emergence of certain mutations may cause HBV reactivation and subsequent ACLF development.

Overall there are few studies, and conflicting data on HBV genetic profiles in P/S and BCP/PC regions in CHB-ACLF patients compared with non-ACLF CHB patients. The aim of our study is to compare HBV genetic features by clonal sequencing analysis of HBV P/S and BCP/PC regions between a cohort of CHB patients with ACLF compared to non-ACLF/CHB cases to further investigate the role of viral genetic factors in CHB disease severity.

## Methods

### Patients, clinical and laboratory tests

In total, 12 ACLF in CHB patients (11/12 male, 12 Chinese, median age 52 yr., range 21–64), were either retrospectively (LF # 1, 2, 3, 4, 5) or prospectively (LF # 13, 14, 15, 16, 17, 18, 19) recruited from Beijing Youan Hospital, Capital Medical University (CMU). All subjects provided written informed consent under an approved Beijing Youan Hospital (Ethics ID LL-2007-002S) institutional ethics board protocol, according to the World Medical Association Declaration of Helsinki. Moreover, sequence data from a cohort of 27 treatment-naïve CHB patients in different clinical immune phases (i.e., immune tolerance, immune active, inactive and HBeAg negative hepatitis phases) was included in the analysis of current study [23, 24].

The clinical and demographic data of all cases collected included age, sex and ethnicity, HBV plasma viral load by clinical PCR assay (i.e., Abbott Molecular *m*2000 RealTI*M*e System, Abbott Park, Illinois, USA, lower limit of detection (LLOD) 10 IU/mL or ~ 50 copies/mL at Calgary Liver Unit and Roche COBAS Ampliprep/COBAS Taqman, Pleasanton, CA, USA, lower limit of detection 20 IU/mL or ~ 100 copies/mL), alanine aminotransferase (ALT), quantitative HBsAg level (qHBsAg, Abbott Diagnostics, Abbott Park, Illinois, USA, sensitivity <=0.05 IU/mL or Roche Diagnostics/Elesys HBsAg Π assays, Mannheim, Germany, sensitivity of <=0.05 IU/mL), HBeAg and antibody to HBeAg (anti-HBe) (Abbott Diagnostics, Mississauga, ON, Canada or Roche Diagnostics/Elecsys HBeAg assays, Mannheim, Germany).

HBV genotype was determined by line probe assay (INNO-LiPA, Innogenetics N. V., Ghent, Belgium at Calgary Liver Unit or by PCR fluorescence assay (specific for genotype B and C, Shanghai Fu Xing Chang Zheng, China). The ACLF in CHB subjects were assessed according to the Asian Pacific Association for the Study of Liver Disease for ACLF diagnosis and management [1]. All subjects were initiated on NA therapy with either lamivudine (300 mg/day, *N* = 2) or entecavir (0.5 mg/day, *N* = 11) after admission to Beijing Youan Hospital.

### Biological sample collection, total DNA isolation and detection of HBV genome in plasma

In total, about 50 mL of EDTA whole blood was collected from each patient. Plasma was isolated by centrifugation and stored at −80 °C. Total DNA was isolated from 500 μL plasma by standard phenol-chloroform/ethanol precipitation. The DNA isolations were carried out in parallel with negative controls that included water or from plasma of a HBsAg-negative healthy individual (designated the mock extraction control).

HBV DNA was detected by nested PCR in plasma using HBV P/S and BCP/PC gene specific primers, respectively. The HBV P/S gene was amplified using previously published primer and conditions [23]. The primer sequences for the first-round HBV BCP/PC gene PCR were direct BCP/PC forward (5′-GCATGGAGACCACCGTGAAC-3′) and direct BCP/PC reverse (5′-ggaaagaagtcagaaggcaa-3′); and nested BCP/PC forward (5′-CATAAGAGGACTCTTGGACT-3′) and nested BCP/PC (5′-GGCAAAAAAGAGAGTAACTC-3′) for the second round. The amplification was performed under conditions below: 94 °C for 40s, 55 °C for 30s, 72 °C for 50 s, 39 cycles total. All experiments included parallel mock nucleic acid isolations and PCR water negative controls. Positive controls included total DNA isolated from liver tissue of a known HBsAg positive individual or in-house HBV plasmid DNA (HBV genotype A full genome). The PCR products were gel purified (GenElute Gel Extraction kit, Sigma-Aldrich, Oakville, ON, Canada).

### Clonal sequence analysis of HBV overlapping polymerase (P)/surface (S) region and basal core promoter (BCP)/precore (PC) regions

HBV P/S gene fragment in 7/12 treatment naïve cases (LF #3, 5, 13, 14, 16, 17 and 19) and BCP/PC gene fragment in 12/12 subjects were cloned using pGEM®-T Easy Vector Systems (Promega, Madison, USA), followed by plasmid isolation (GenElute Plasmid Miniprep Kit, Sigma-Aldrich, Oakville, ON, Canada) according to manufacturer’s instructions. A median 17 clones/sample was sequenced bi-directionally with universal primers at 3730 XL sequencing system (Applied Biosystem, Foster City, CA, USA) and sequences were analyzed with Geneious 7.1.9 and MEGA version 6.06. HBV diversity (distance ± SEM) of HBV P/S and BCP/PC gene sequences of CHB/ACLF patients were compared to previously published sequence data from 27 treatment-naïve CHB patients of different immune phases [23] by sequences alignment and within group diversity analysis. The HBV P/S clonal sequences were aligned using Genbank reference sequences published previously [23], translated for S and P protein and aligned with sequences of the same genotype to determine putative nongenotypic substitutions. For all clones, aa 61–250 of the RT/P and aa 100–200 of the S antigenic determinant region were analysed. The G1896A mutation in PC region and A1762T/G1764A dual mutations in BCP/PC region were analysed in HBV BCP/PC clones of different groups.

### Data analysis

An analysis of variability (ANOVA) with appropriate post hoc test was used for the comparison of continuous data and the chi-square or Fisher’s exact test was used for the comparison of categorical data. Two-tailed *p* values of <0.05 were considered statistically significant. Statistical analysis was performed with Prism 6.0.

## Results

### Demographic, serological, and histological data of CHB patients

The clinical data of study subjects is summarized in Table 1. In total, 7 treatment naive CHB, 5 NA-experienced cases with ACLF were enrolled (median age 52 yrs., 11/12 M, 6/12 HBeAg positive) (Additional file 1: Table S1). At the time of hospital presentation and sample collection all patients were not on active NA therapy (5 cases LF # 1, 2, 4, 15 and 18 had stopped treatment median 5 months previously). 8/12 patients had underlying cirrhosis according to the results of abdominal ultrasound. The overall median serum HBV viral load of all ACLF patients was 4.7 log IU/mL (range 2.2–7.3, data not shown). The median serum HBV viral load was log 5.7 IU/mL (range 4.3–7.3) and log 3.7 IU/mL (range 2.2–5.9) of HBeAg positive and HBeAg negative CHB-ACLF patients, respectively. All ACLF cases were found to have HBV reactivation as the cause of ACLF. Co-infection with hepatitis A, Delta, C and E was excluded. HBV genotype test (done by clinical assay) was available in 8/12 cases and showed that 3 were HBV genotype B vs. 5 genotype C. 4/12 patients died within 4 weeks of presentation, and 8/12 patients who received rescue NA therapy survived at week 4 of follow-up from onset of liver failure, with 5/12 decline in HBV DNA and ALT and improved liver function.

**Table 1:** Demographic and clinical data of Chronic Hepatitis B (CHB)-associated Acute-on-Chronic Liver Failure (ACLF) patients (*N* = 12), treatment naive CHB patients (NA = 27)

	CHB-ACLF (HBeAg positive, *N* = 6)	CHB-ACLF (HBeAg negative, *N* = 6)	Treatment naïve CHB			
			Immune tolerance (*N* = 2)	Immune active (*N* = 9)	Inactive (*N* = 5)	HBeAg negative hepatitis (*N* = 11)
Median age (range)/# male	49 (21–64)/6	52 (26–57)/5	28 (20–35)/1	47 (23–50)/2	52 (45–61)/1	43 (36–57)/11
Ethnicity	6 Chinese	6 Chinese	1 Chinese; 1 Eastern European	6 Chinese; 1 Tibetian; 1 Vietnam; 1 Indonesia	3 East- Europe; 1 Korean; 1 African	6 Chinese; 1 Lebanese; 2 Vietnam; 1 Fillipino; 1 Egyptian
Median HBV DNA (rang, log IU/mL)	5.7 (4.3–7.3)	3.7 (2.2–5.9)	8.5 (8.0–8.6)	8.0 (4.5–9.0)	3.1 (UD-3.6)	4.3 (2.9–6.2)
Median ALT (rang, U/L)	562 (23–1584)	112 (48–2347)	37 (20–33)	86 (27–353)	25 (14–48)	48 (20–106)
# HBeAg negative patients	0/6	6/6	0/2	0/9	5/5	11/11
Genotype	1 B; 3 C; 2 unknown	2 B; 2 C; 2 unknown	1 B, 1 D	3 B, 5 C, 1 D	1 A, 1 B, 1 C, 1 D, 1 E	4 B, 4 C, 2 D, 1E
Median HBsAg (rang, log IU/mL^3^)	unknown	unknown	unknown	4.0 (2–9.8)	3.9 (2.7–5.1)	3.5 (1.6–9.1)
# Liver fibrosis	3/6	5/6	0/2	3/9	0/5	5/11
# History of NA treatment	1 LAM	4 LAM	N/A	NA	N/A	NA
# Surviral (4 weeks after onset)	2/6	2/6	All alive	All alive	All alive	All alive

*UD* undetectable

### ACLF in CHB patients carry more mutations at residues associated with immune escape (IE)

**Fig. 1:**
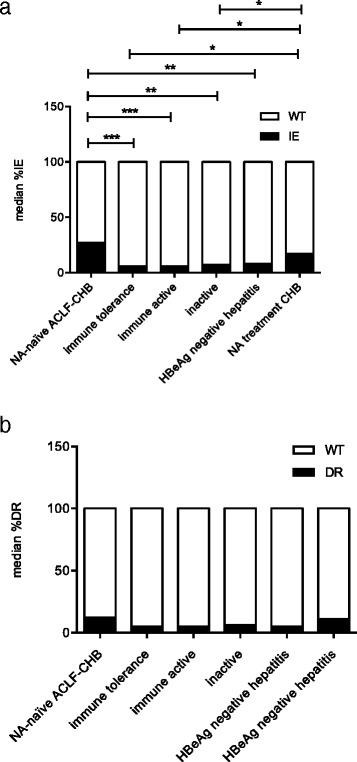
Frequency of HBV immune escape (IE) and drug resistance (DR) mutations in plasma of NA-treatment naïve CHB patients with (N = X) or without ACLF (N = X). **a** The incidence of minor IE mutations was higher in NA-treatment naïve ACLF-CHB patients than in treatment naïve CHB patients of different immune phases. * *p* < 0.05*,* **p < 0.005, ***p < 0.001. **b** Minor DR mutations were present in patients of all different groups. No significant difference of DR incidence was found between groups

In 7/12 NA-treatment naïve ACLF / CHB patients, the HBV P/S gene fragment was amplified by nested PCR (LF #3, 5, 13, 14, 16, 17 and 19). The incidence of HBV mutations at positions associated with immune escape (IE) and drug resistance (DR) between CHB patients with ACLF versus treatment naïve CHB patients of different phases was compared. The incidence of IE mutants was higher in ACLF patients (median 27%) than in treatment naïve CHB subjects of different clinical phases (median 6%–8%) (Fig. 1a). However, minor HBV DR mutations were found with comparable frequency among all patient groups (*p* > 0.05; Fig. 1b). Clusters of mutations between aa 100–200 of S gene were observed in all 7 ACLF in CHB patients (Additional file 1: Table S2-S3, Figure S2).

### Higher incidence of A1762T/G1764A and G1896A mutations in HBV BCP/PC region in HBeAg negative hepatitis B patients

**Fig. 2:**
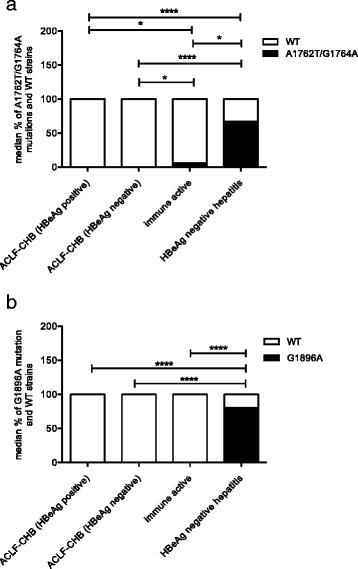
The median incidence of A1762T/G1764A dual mutations in HBV basal core promoter (BCP) region and G1896A mutation in HBV precore (PC) region among different groups (ACLF-CHB, immune active & HBeAg negative hepatitis CHB groups). **a** The incidence of A1762T/G1764A dual mutations was significantly higher in HBeAg negative hepatitis group than in both HBeAg positive (****p < 0.0001) and HBeAg negative ACLF-CHB groups (****p < 0.0001) and immune active group (*p < 0.05). The dual mutations were also more frequently seen in immune active group than in both ACLF-CHB group (*p < 0.05). **b** The incidence of G1896A mutation in HBV PC region was significantly more frequent in HBeAg negative hepatitis group than in both ACLF-CHB groups (****p < 0.0001) and immune active group (****p < 0.0001)

The HBV BCP/PC gene fragment was amplified in 12/12 ACLF in CHB patients, and mutations therein compared between HBeAg positive (*N* = 6) and HBeAg negative (N = 6) groups, respectively. The median frequency of A1762T/G1764A dual mutations in BCP region was significantly higher in HBeAg negative hepatitis CHB (67%, **** *p* < 0.0001) as well as the immune active CHB group (6%, *p < 0.05) compared to the ACLF-CHB cohort (both 0% in both HBeAg positive and HBeAg negative) (Fig. 2a). In addition, the A1762T/G1764A mutations were more frequently seen in immune active CHB group than in both ACLF-CHB groups (*****p < 0.0001; Fig. 2a). Similarly, the incidence of classic G1896A mutation in HBV precore region was significantly higher in HBeAg negative hepatitis group (80%) than that in both ACLF-CHB groups (0%, ****p < 0.0001) and immune active group (0%, ****p < 0.0001; Fig. 2b). HBV genetic diversity in both P/S and BCP/PC regions did not demonstrate a significant difference among different groups (Additional file 1: Figure S1a-b).

## Discussion

Acute reactivation of chronic hepatitis B leading to ACLF is a rare clinical entity frequently leading to a life-threatening liver disease with poor survival without liver transplant. The genetic characteristics and dynamics of HBV quasispecies may reflect distinct host immune responses, and disease courses. However, there is limited and conflicting data on the role of specific HBV variants in the pathogenesis of HBV-related fulminant liver failure. In the present study, the presence of certain mutations and diversity of HBV P/S and BCP/PC gene in plasma of ACLF-CHB patients was compared to sequence data from CHB patients [23] to determine if there were specific genetic profile (i.e., mutations) associated with disease severity. We found that the ACLF patients enrolled were mainly HBV genotype C (5/8) and 6/12 were HBeAg positive. A distinct BCP/PC mutation was not characteristic for HBV genomes in CHB patients with ACLF. However, various mutations clustering at aa 100–200 of HBV S gene were more common in ACLF-CHB patients. Moreover, the incidence of S gene IE associated mutations was significantly higher in ACLF-CHB patients compared to treatment naive non-ACLF CHB patient of different immune phases. In a recent study, Salpini et al. reported 75.9% of HBV reactivated patients having more than one HBsAg mutations and most of them (8/13) were located in the major hydrophilic loop [25], which is similar to what we observed in the ACLF-CHB patients due to HBV reactivation.

The dominance of HBV genotype C in our ACLF-CHB cohort suggested that genotype C might be associated with increased risk of ACLF development. This data is in agreement with some studies in which genotype C (compared to HBV genotype B), was associated with a higher prevalence of HBeAg, higher HBV DNA levels, more aggressive histological activity, as well as severe acute exacerbation and increased risk of HBV reactivation [26–34]. For instance, a study from Taiwan reported genotype C was significantly more frequent in patients with multiple episodes of acute exacerbation who failed to have HBeAg seroconversion compared with other patients [27]. Additionally, all of the cross-sectional studies from endemic areas in Asia demonstrate that genotype C is associated with more advanced liver disease than genotype B [26, 35–38]. However, in other reports either no significant difference between genotype B vs C was found in association with acute CHB exacerbation [39, 40], or severe icteric acute exacerbation was associated with genotype B rather than genotype C [9, 41].

The G1896A precore mutation was reported in association with fulminant hepatitis B in patients from Japan [11, 13, 42–44] Israel [12], and southern Mediterranean countries [45], and the A1762T/G174A mutations in BCP region was frequently showed in Japanese cohort [16]. However, there have been conflicting reports on the association between specific mutations in the HBV genome and fulminant hepatitis, especially the G1896A and A1762T/G1764A mutations [11, 12, 16, 19, 46]. In our study the frequency of G1896A and A1762T/G1764A mutations was significantly greater in patients from HBeAg negative hepatitis groups compared to CHB patients with ACLF. Our data also demonstrated no significant difference of diversity in HBV P/S and BCP/PC gene in patients from ACLF-CHB groups compared with other groups.

The emergence of S viral variants with altered biological function have been implicated in the pathogenesis of ACLF. The variants can be cytotoxic due to enhanced protein expression or lead to altered immunogenicity, resulting in immune escape or robust immune response. Specific S gene mutations or a combination of all S gene mutants were found to be responsible for viral secretion defect or even completely blocked viral particle secretion in in vitro functional studies [21].

Minor drug resistant (DR) mutations in HBV P gene were found in all patient groups sequenced with no inter-group significant difference noted. Further no detectable NA resistance (i.e., YMDD (tyrosine-methionine-aspartate-aspartate) mutation) was noted in any of the ACLF-CHB patient by clinical assay (data not shown). This indicated the emergence of DR mutants and subsequent viral breakthrough was not the cause of ACLF occurrence in our patient cohort (even despite previous exposure to NA therapy). As expected, acute hepatic flare in 8/12 cases with underlying cirrhosis precipitated decompensation. It should be noted that according to APASL the term “acute on chronic liver failure” is used for chronically HBV infected patients regardless of whether they have cirrhosis or not. However, the US Acute Liver Failure study group and the American Association for the Study of Liver Disease differentiates between HBV infected patients that develop ALF (i.e, if cirrhotic designated as HBV-ACLF vs. non-cirrhotic designated as HBV-ALF). However, for the purpose of this study we used the APASL guidelines for the definition of ACLF, for all 12 patients (8/12 cirrhotic) enrolled.

The study was limited by sample size with only 12 patients with ACLF. Further, assessment of HBV in liver tissue, longitudinal follow-up and NGS of HBV full genome sequences could provide greater details on HBV quasispecies. However, ACLF is a rare and devastating clinical entity of which there is limited published systematic analysis of HBV variants.

In summary, the pathogenesis of fulminant hepatitis B has been linked to three main virological markers as (i) an increased viral replication, (ii) a change of viral gene expression and/or (iii) an alteration of B and T cell epitopes [47].

## Conclusions

Our study demonstrated greater incidence of IE mutations and clusters of variants in HBV S genes were present in ACLF-CHB patients compared to other non-ACLF CHB patient. Interestingly, the G1896A and A1762T/G1764A mutations, although associated with fulminant hepatitis B development in other studies, were found at low frequency in ACLF patients compared to non-ACLF/HBeAg negative hepatitis CHB patients. Additional studies including functional assessment of HBV variants using in vitro models as well as analysis of host factors (i.e., HBV specific immune responses) will help increase our understanding of the mechanisms of development of ACLF in CHB patients.

## Additional file.

Additional file 1: Table S1. Clinical and virological data of 12 patients with Chronic-Hepatitis B (CHB)-associated Acute-on-Chronic Liver Failure (ACLF). **Table S2.** The substitutions between aa 100–200 region of HBV S gene in plasma from in Chronic Hepatitis B patients with Acute/Chronic Liver Failure. **Table S3.** Summary of classic IE mutations and list of classic IE mutations and other mutations in “a determinant” region of HBV S gene found in Chronic Hepatitis B patients with Acute/Chronic Liver Failure (*N* = 7). **Figure S1.** Comparison of HBV P/S and BCP/PC sequences diversity in CHB patients with and without Acute-on-Chronic Liver Failure (ACLF). (a) Analysis of HBV P/S sequence diversity in plasma among different groups. The viral diversity of P/S sequences was comparable between each group. (b) Analysis of HBV BCP/PC sequence diversity in plasma of different groups. There was no significant difference in HBV diversity of BCP/PC gene observed among distinct groups. **Figure S2.** Alignment of HBV S gene consensus sequences within the “a” determinant region to HBV sequences from a cohort of chronic HBV patients with Acute/Chronic Liver Failure (*N* = 7). The classic IE mutations in the patients were indicated by red arrows while other substitutions are noted by black arrows. Both classic IE-associated and other substitutions in “a” determinant are common among these patients. A specific list of substitutions for each patient is provided in Additional file 1: Table S3. (DOCX 588 kb)

## Abbreviations

ACLF: Acute on chronic liver failure
ALT: Alanine transaminase
BCP: Basal core promotor
CHB: Chronic hepatitis B
DR: Drug resistant
HBeAg: Hepatitis B e antigen
HBV: Hepatitits B virus
IE: Immune escape
NA: Nucleot/side analogue
NGS: Next generation sequencing
P: Polymerase
PC: Precore
RT: Reverse transcriptase
S: Surface
