# Supplementary material for: Chronic hepatitis B carriers with acute on chronic liver failure show increased HBV surface gene mutations, including immune escape variants
Source: Virol J. 2017 Oct 24;14:203. doi: 10.1186/s12985-017-0870-x (PMC5655973; doi:10.1186/s12985-017-0870-x)
Supplement: Additional file 1: Table S1. — Clinical and virological data of 12 patients with Chronic-Hepatitis B (CHB)-associated Acute-on-Chronic Liver Failure (ACLF). Table S2. The substitutions between aa 100–200 region of HBV S gene in plasma from in Chronic Hepatitis B patients with Acute/Chronic Liver Failure. Table S3. Summary of classic IE mutations and list of classic IE mutations and other mutations in “a determinant” region of HBV S gene found in Chronic Hepatitis B patients with Acute/Chronic Liver Failure (N = 7). Figure S1. Comparison of HBV P/S and BCP/PC sequences diversity in CHB patients with and without Acute-on-Chronic Liver Failure (ACLF). (a) Analysis of HBV P/S sequence diversity in plasma among different groups. The viral diversity of P/S sequences was comparable between each group. (b) Analysis of HBV BCP/PC sequence diversity in plasma of different groups. There was no significant difference in HBV diversity of BCP/PC gene observed among distinct groups. Figure S2. Alignment of HBV S gene consensus sequences within the “a” determinant region to HBV sequences from a cohort of chronic HBV patients with Acute/Chronic Liver Failure (N = 7). The classic IE mutations in the patients were indicated by red arrows while other substitutions are noted by black arrows. Both classic IE-associated and other substitutions in “a” determinant are common among these patients. A specific list of substitutions for each patient is provided in Additional file 1: Table S3. (DOCX 588 kb) [file 12985_2017_870_MOESM1_ESM.docx]

**Additional file 1: TABLES AND FIGURE**

Table S1 Clinical and virological data of 12 patients with Chronic-Hepatitis B (CHB)-associated Acute-on-Chronic Liver Failure (ACLF).

|  | Age/  Sex | ALT (U/L) | T-bil  (μmol/L) | PLT  (10^9/L) | PTA% | HBV DNA  (log IU/mL) | HBeAg/anti-HBeAg | Genotype | Liver cirrhotic signs by ultrasound | Previous NA therapy (NA/mo) | Clinical Outcome (4 weeks after admission) |
| --- | --- | --- | --- | --- | --- | --- | --- | --- | --- | --- | --- |
| LF1 | 57/F | 553 | 393 | 161 | 7 | 3.5 | Neg/pos | B | No | LAM/6 | Survived |
| LF2 | 49/M | 60 | 243 | 113 | 38 | 3.3 | Neg/pos | B | Yes | LAM/5 | Survived |
| LF3 | 21/M | 796 | 808 | 101 | 35 | 5.6 | Pos/neg | C | Yes | N/A | Survived |
| LF4 | 50/M | 2347 | 205 | 99 | 29 | 5.9 | Neg/pos | UN | Yes | LAM/8 | Died |
| LF5 | 28/M | 1584 | 249 | 83 | 17 | 7.3 | Pos/neg | B | Yes | NA | Survived |
| LF13 | 39/M | 23 | 294 | 167 | 39 | UN | Pos/neg | UN | No | NA | Survived |
| LF14 | 58/M | 35 | 565 | 95 | 30 | 4.8 | Pos/neg | C | Yes | NA | Died |
| LF15 | 64/M | 1145 | 301 | 139 | 28 | 5.8 | Pos/neg | UN | Yes | LAM/3 | Died |
| LF16 | 56/M | 48 | 322 | 244 | 38 | 3.8 | Neg/pos | C | No | NA | Survived |
| LF17 | 61/M | 328 | 269 | 20 | 31 | 4.3 | Pos/neg | C | Yes | NA | Survived |
| LF18 | 26/M | 81 | 492 | 57 | 18 | 2.2 | Neg/pos | UN | Yes | LAM/5 | Died |
| LF19 | 54/M | 142 | 289 | 423 | 20 | 4.7 | Neg/pos | C | No | NA | Survived |

ALT: alanine amino transaminase; T-Bil: total bilirubin; PLT: platelet; PTA: prothrombin activity NA: nucleos/tide analogue; Neg: negative; Pos: positive.

Table S2 The substitutions between aa 100-200 region of HBV S gene in plasma from in Chronic Hepatitis B patients with Acute/Chronic Liver Failure.

| ID | # Clones | Genotype | HBsAg (aa 100-200)  (# of clones) |
| --- | --- | --- | --- |
| LF #3 | 22 | C | Q101H(8)  M103L(1)  V106I(1)  L109P(1)  L110P(1)  S117N(1)  T/I126S(10)  Q129L(1)  S132P(1)  N146I(1)  T148A(1)  A167S(1)  S171P(2)  P178Q(1)  F179L(1)  L186P(1)  S187P(1)  W197R(1)  M198T(1)  W199R(1) |
| LF #5 | 17 | B | K22N (1)  T126A (15)  K141R (1)  K122E (1)  F134L (1)  G145R (1)  L186P (1)  R169S (1)  L176P (1)  G181R (1)  F183Y/S (2)  C138S (1)  W156R (1)  F164G (1)  S117N (1)  W191R (1)  C107R (1)  W182R (1) |
| ID | # Clones | Genotype | HBsAg (aa 100-200)  (# of clones) |
| LF #5 | 17 | B | N146S (1)  F183S (1)  S193P (1)  L192P (1)  Y100C (1)  S171P (1) |
| LF #13 | 17 | D | T116A(1)  T/P127A(1)  S143P(1)  F161L(1)  E164G(1)  S167P(1)  A168V(1)  F179L(1)  F183S/I(2)  I195V(1) |
| LF #14 | 15 | C | G101R (13)  C39W (1)  T140I (1)  K141R (1)  V180A (1)  L110P (1)  R160stop (1)  C149Y (1)  F170S (1)  W163stop (1)  F134S (1)  A157X (1)  C124X (1)  S117N (1)  C149S (1)  F179S (1)  M197V (1)  T113I (1)  S114P (1)  S193P (1) |
| LF #16 | 17 | C | F134L (1)  C149R (1)  A159T (1)  M197V (1)  G130N (11)  W36stop (1) |
| ID | # Clones | Genotype | HBsAg (aa 100-200)  (# of clones) |
| LF #16 | 17 | C | C137R (1)  W196stop (1)  V177E (1)  C/R124Y (1)  F170L (1)  K141E (1)  M198V (1)  S155P (1)  W172stop (1) |
| LF #17 | 18 | C | T131I (9)  L175X (1)  I195T (1)  S143P (1)  W156stop (4)  I126T (1)  N146D (1)  W156R (1)  C121R (1)  Q181stop (1)  V180A (1)  G145E (1)  P153L (1)  F170L (1)  L173P (1)  I152T (1)  S154L (1)  M198V (1)  F158S (1)  F183L (1)  T118A (1)  R169C (1) |
| LF #19 | 17 | C | P111X (1)  T189I (2)  L110P (1)  S114P (1)  S117N/G (2)  S136F (1)  T140A (1)  A159E (1)  R160G (1)  V180A (1)  L21stop (4) |
| ID | # Clones | Genotype | HBsAg (aa 100-200)  (# of clones) |
| LF #19 | 17 | C | C107R (1)  F170L (1)  T189I (3)  I195V (1)  T189I (4)  K141T (1)  F158L (1)  F134L (1)  G145E (1)  Y200C (1)  L176P (1)  W182R (1)  D144G/N (2)  W199R (1)  L104W (1)  P120L (1)  C124R (1)  A159E (1)  W172stop (1) |

Table S3 Summary of classic IE mutations and list of classic IE mutations and other mutations in “a determinant” region of HBV S gene found in Chronic Hepatitis B patients with Acute/Chronic Liver Failure (N=7)

| ID | Classic IE mutations | Other mutations | List of classic IE mutations in “a determinant” region of HBV S gene |
| --- | --- | --- | --- |
| LF #3 | T/I126S, Q129L | Q101H, M103L, V106I, L109P, L110P, S117N, S132P, N146I, T148A | sG145R^*$^(1-10), T116N^*(^11), sK141E^*^(6, 12, 13), sD144A/E^*#(^9, 12, 14, 15), , P120S/E^*^(7, 8, 14), I/T126A/N/I/S^*^(9, 12-14, 16), sQ129H/R^*^(12, 15, 17), sM133L^*#^(15, 17), sP142S^*^(8, 9, 12, 13) |
| LF #5 | T126A, K141R, G145R | K122E, F134L, C148S, C107R |  |
| LF #13 | T116A | T/P127A, S143P, F161L |  |
| LF #14 | K141R | G101R, T140I, L110P, R160Stop, C149Y, F134S, A157X, C124X, S117N, C149S, T113I, S114P |  |
| LF #16 | K141E | F134L, C149R, A159T, G130N, C137R, C/R124Y, S155P |  |
| LF #17 | I126T, G145E | S143P, N146D, W156R, C121R, P153L, I152T, S154L, F158S, T118A |  |
| LF #19 | G145E, D144G/N, P120L | P111X, L110P, S114P, S117N/G, S136F, T140A, A159E, R160G, C107R, F158L, F134L, L104W, C124R, A159E |  |

* Immune escape mutation; # Vaccine escape mutation; $ Occult hepatitis B associated mutation.

Figure S1 Comparison of HBV P/S and BCP/PC sequences diversity in CHB patients with and without Acute-on-Chronic Liver Failure (ACLF).

(a) Analysis of HBV P/S sequence diversity in plasma among different groups. The viral diversity of P/S sequences was comparable between each group.

(b) Analysis of HBV BCP/PC sequence diversity in plasma of different groups. There was no significant difference in HBV diversity of BCP/PC gene observed among distinct groups.

Figure S2. Alignment of HBV S gene consensus sequences within the “a” determinant region to HBV sequences from a cohort of chronic HBV patients with Acute/Chronic Liver Failure (N=7). The classic IE mutations in the patients were indicated by red arrows while other substitutions are noted by black arrows. Both classic IE-associated and other substitutions in “a” determinant are common among these patients. A specific list of substitutions for each patient is provided in Table S3.
